# Single-cell resolved ploidy and chromosomal aberrations in nonalcoholic steatohepatitis-(NASH) induced hepatocellular carcinoma and its precursor lesions

**DOI:** 10.1038/s41598-022-27173-z

**Published:** 2022-12-31

**Authors:** Juliane Friemel, Irianna Torres, Elizabeth Brauneis, Tim Thörner, Alejandro A. Schäffer, E. Michael Gertz, Tobias Grob, Kati Seidl, Achim Weber, Thomas Ried, Kerstin Heselmeyer-Haddad

**Affiliations:** 1 Genetics Branch, CCR, National Cancer Institute, NIH, Bethesda, MD USA; 2 Department of Pathology and Molecular Pathology, University and University Hospital Zurich, Zurich, Switzerland; 3 Cancer Data Science Laboratory, CCR, National Cancer Institute, NIH, Bethesda, MD USA; 4 Computational Biology Branch, National Center for Biotechnology Information, National Library of Medicine, NIH, Bethesda, MD USA; 5 Department of Pathology, University of Bern, Bern, Switzerland

**Keywords:** Cancer, Medical research

## Abstract

Nonalcoholic steatohepatitis (NASH)-induced hepatocellular carcinoma (HCC) and its precursor, nonalcoholic fatty liver disease (NAFLD) are an unmet health issue due to widespread obesity. We assessed copy number changes of genes associated with hepatocarcinogenesis and oxidative pathways at a single-cell level. Eleven patients with NASH-HCC and 11 patients with NAFLD were included. Eight probes were analyzed using multiplex interphase fluorescence in situ hybridization (miFISH), single-cell imaging and phylogenetic tree modelling: Telomerase reverse transcriptase (*TERT*), C-Myc (*MYC*), hepatocyte growth factor receptor tyrosine kinase (*MET*), tumor protein 53 (*TP53*), cyclin D1 (*CCND1*), human epidermal growth factor receptor 2 (*HER2*), the fragile histidine triad gene (*FHIT*) and FRA16D oxidoreductase (*WWOX*). Each NASH-HCC tumor had up to 14 distinct clonal signal patterns indicating multiclonality, which correlated with high tumor grade. Changes frequently observed were *TP53* losses, 45%; *MYC* gains, 36%; *WWOX* losses, 36%; and *HER2* gains, 18%. Whole-genome duplications were frequent (82%) with aberrant tetraploid cells evolving from diploid ancestors. Non-tumorous NAFLD/NASH biopsies did not harbor clonal copy number changes. Fine mapping of NASH-HCC using single-cell multiplex FISH shows that branched tumor evolution involves genome duplication and that multiclonality increases with tumor grade. The loss of oxidoreductase *WWOX and HER2* gains could be potentially associated with NASH-induced hepatocellular carcinoma.

## Introduction

The incidence of nonalcoholic fatty liver disease (NAFLD) or nonalcoholic steatohepatitis (NASH) induced hepatocellular carcinoma (HCC) is increasing in countries with endemic metabolic syndrome and obesity^1–3^. Specific patient characteristics have have been reported including older age, higher burden of metabolic risk factors, and HCC development in non-cirrhotic livers^4,5^. On a cellular level, it is still poorly understood how aberrant metabolism and inflammation contribute to DNA instability and cancer^6^.

Established risk factors for hepatocellular carcinoma development are partially associated with etiology-specific mutations such as *CTNNB1* (oral contraception), *TP53* (aflatoxin), *HFE1* (alcohol abuse), chromosome 1q gain (hepatitis C) and mutagenesis through insertion of oncoproteins (hepatitis B)^7–9^. Non-etiology-specific alterations involve *TERT*, which is located at chromosome band 5p15.33. Point mutations within the promotor region of *TERT* are frequent and have been associated with HCC among patients with metabolic syndrome, but also with alcohol intake and with hepatitis C infection^10^. Gains of *MYC*, *CCND1*, *MET* and *HER2* have been detected by comparative genomic hybridization (CGH) or FISH^11–13^. Frequent losses comprise chromosome 17p (*TP53*), 4q (*ING2*) and 8p (*DLC-1*) in 26–31% of HCC cases^14^. Two targets covering oxidative cell stress pathways have been described in solid tumors: *FHIT* and *WWOX* are each located in a prominent fragile site susceptible to DNA damage in liver cells. *FHIT* (3p14.2) is involved in purine metabolism and is altered in hereditary clear cell renal cell carcinoma and small cell lung cancer^15,16^. *WWOX* (16q23.1) encodes for a member of short-chain dehydrogenases; in genome-wide association studies and eQTL studies, variants in or near *WWOX* and reduced expression of *WWOX* were associated with familial dyslipidemia and metabolic syndrome^17^. Losses were found in HCC cell lines, but not in patient material with specific NASH etiology^18^.

Among the variety of clonal and subclonal mutations or aneuploidy in hepatocarcinogenesis, polyploidization has been suggested as a driver of tumorigenesis and as a biomarker associated with *TP53* mutations in a study by Bou-Nader et al.^19^ Two types of polyploidization have been observed in the liver: cellular polyploidy (binuclear hepatocytes) considered as physiological event and nuclear polyploidy (DNA content per nucleus, 2n, 4n, 8n) possibly a pathological event and promoted by endoreplication^20^. In nonalcoholic fatty liver disease of rodents, Gentric et al. showed increased mononuclear polyploid cells^21^. These findings are in agreement with earlier cell culture studies where polyploidy of hepatocytes was observed in response to cell stress^22,23^. The so called “ploidy conveyor”, was proposed as a hypothesis involving polyploidization of hepatocytes during lifetime as an adaptation to (and protection from) cell damage. Polyploidy may allow hepatocytes to increase liver specific functions while buffering against genomic damage^24,25^. The question of whether polyploidy protects the cell or is by itself a carcinogenic factor is unsolved. These are not mutually exclusive possibilities since liver cancer onset typically occurs after the age of human reproduction, when evolutionary selection for favorable traits diminishes. Recent work by Matsumoto et al. showed that polyploid hepatocytes are prone to genomic damage because they undergo ploidy reduction right before initiation of carcinogenesis^26^. A study by Zhang et al. demonstrated that the polyploid state plays a a tumor-suppressive role in the liver^27^.

Our aim was to trace carcinogenesis, i.e. evolution of aneuploidy and polyploidy in nonalcoholic steatohepatitis (NASH)-induced hepatocellular carcinoma (HCC) and its precursor, nonalcoholic fatty liver disease (NAFLD). We therefore used the novel single-cell miFISH approach which is applied to monolayered preparations of intact single nuclei derived from thick sections of archival patient material, thereby avoiding truncation artifacts and overlapping nuclei that hamper accurate signal number enumeration in conventional tissue FISH. Multiplexing FISH signals for ten genomic loci, including two centromere control probes, allows for the simultaneous assessment of nuclear ploidy and exact copy number changes for eight genes relevant for liver carcinogenesis within intact individual nuclei giving new insights into the development of tumor clonality, heterogeneity and polyploidization in NASH-induced liver cancers and their potential NAFLD/NASH precursor lesions.

## Materials and methods

### Patients and samples

A total of 11 patients with NASH-HCC liver resection and liver biopsies of 11 patients with NAFLD were analyzed (mean age 68 ± 7 and 46 ± 14). Formalin-fixed, paraffin-embedded tissue samples, retrieved from the archives of the Department of Surgical Pathology, University Hospital Zurich, were included if (global) liver steatosis (fat > 5%) was present and clinical records yielded negativity for hepatitis serology testing and self-reported alcohol consumption of less than the equivalent of 18 ml of pure alcohol per day, which generally corresponds to a bottle of beer or a glass of wine. A history of diabetes, metabolic syndrome or obesity was taken as additional but not mandatory criteria.

Among hepatocellular carcinoma patients the male:female ratio was 10:1 with a mean tumor size of 9.3 ± 4 cm (Table 1). Tumor grades and stages were determined by the WHO classification^28^, vascular invasion was present in the majority of cases (9/11). Macrosteatosis was not so frequent, percentages (abundance of lipid-laden hepatocytes/total number of hepatocytes) ranged between 5 and 40% (mean 22%), which is a known phenomenon of burned out fatty liver disease in manifest hepatocellular carcinoma. Among the NAFLD patients, the male:female ratio was more evenly distributed (6:5). Nonalcoholic fatty liver disease activity scores (NAS), a composition of steatosis, lobular inflammation and ballooning, were grouped into categories 1–2 (n = 2 patients), 3–4 (n = 5 patients) and 5 (n = 4 patients)^29,30^. Per definition 3–4 represent borderline steatohepatitis (mild form), > 5 is considered as manifested steatohepatitis (progressive form). The highest possible score of 8, i.e., severe disease, was not encountered. Three normal liver biopsies of donor organs (prior to transplant) were used as normal controls. Statistical analysis was performed using graphPad Prism software version 9.4.1 https://www.graphpad.com/scientific-software/prism/.

**Table 1:** Summary of ploidy, numerical aberrations and instability index in NASH-induced hepatocellular carcinoma cases.

No	Age	M:F	Tumor size	Stage grade	Fibrosis^a^	Major clone	Minor clone	Ancestry of clones^b^	Polyploid tumor clones present	Average ploidy^c^	Instability index^d^	Diversification	Mutational status
1	70	m	7	T2G2	3	*MYC* gain*, *MET* gain	*MYC/MET gain, TP53* loss, *HER2* gain	Yes	Yes, subset with equivalent aberrations	3.32	45.8	High	***TERT* c.-124C > T *CTNNB1* S33P**
2	69	m	10.4	T3G3	2	***TP53* loss**, *WWOX* loss	*TP53* loss, *WWOX* loss, *HER2* loss, *TERT* loss	Yes	Yes exclusively, accumulated aberrations	4.05	26.17	High	***TP53* H179R**
3	73	m	14	T4G3	3	*WWOX* loss	***TP53* loss**	No	Yes, < 5%	2.38	36.4	High	***TERT* c.-124C > T**
4	71	m	13	T2G3	2	*HER2* gain, ***MYC* gain**, *TERT* gain	*HER2* gain, *MYC* gain, *TP53* loss	Yes	Yes, < 5%	2.58	33.66	High	***CTNNB1* T41I *TERT* c.-124C > T**
5	69	m	8	T3G2	4	***MYC* gain** 3–4 copies	*MYC* gain *MET* loss	Yes	Yes, major clone 2, accumulated aberrations	2.47	16.7	Low	***TERT* c.-124C > T**
6	77	m	2.9	T1G1	1a	***MYC* gain** 4 copies	*MYC* gain 5 copies	Yes	Yes, < 5%	2.12	15.6	Low	***TERT* c.-124C > T**
7	60	m	18	T1G1	4	*TERT* gain, *MET* gain, *WWOX* loss, ***TP53* loss**	*TERT* gain, *MET* gain, *WWOX* loss	Yes	No	2.07	19.11	High	***CTNNB1* S37A *TERT* c.-124C > T**
8	77	m	9.5	T2G3	3	*HER2* loss, *WWOX* loss	*HER2* loss, *WWOX* loss, *TERT* gain	Yes	No	2.12	27.7	High	***TERT* c.-124C > T**
9	64	f	9.2	T2G3	1a	***TP53* loss**	***TP53* and *MYC* loss**	Yes	Yes, subset with accumulated aberrations	3.17	24.08	High	***TP53* R175H**
10	63	m	4	T2G2	4	***TP53* loss**	*TP53* loss in tetraploid cells	Yes	yes, major clone 2	2.23	6.55	Low	***TP53* S241T *TERT* c.-124C > T**
11	54	m	7	T3G2	4	*CCND1* gain, *TP53* loss	*CCND1* gain, *TP53* loss, *MET* gain (low)	Yes	Yes, < 5%	2.05	14.2	Low	***TERT* c.-124C > T**

^a^Fibrosis stage specific for nonalcoholic fatty liver disease/nonalcoholic steatohepatitis scoring.

^b^Ancestry/relation of clones assessed by phylogenetic tree modelling.

^c^Ploidy calculated from centromere probes of lesional (cancer) cells.

^d^Instability index calculated as number of signal patterns/100 nuclei.

### Ethics statement

Law abidance of this retrospective study was reviewed and approved by the local ethics committee of the Canton of Zurich. The local ethics committee (full name: Kantonale Ethikkommission [KEK], Zurich, Switzerland) waived the informed consent due to the retrospective nature of our study. The accession numbers for this study assigned by the Kantonale Ethikkommision are: StV 26-2005, KEK-ZH-Nr. 2013-0382, PB_2018_00252. All methods were performed in accordance with relevant guidelines and regulations.

### Cytospin preparation

Cytospin preparations of intact nuclei derived from disintegrated archival patient material were performed as described^31^. Briefly, 3 µm formalin-fixed, paraffin-embedded (FFPE) tissue sections stained with Hematoxylin–Eosin were used to evaluate the morphology and outline a representative tumor area (tumor content 80–100%, tumor area 4 cm^2^ for resections specimens or biopsy cores of 1–1.5 cm length). Adjacent 50 µm sections of the FFPE archival patient tissue were cut, the representative area was macro-dissected from the sections and disintegrated with 0.1% protease. The resulting single-cell suspensions were used to prepare cytospins (Shandon Cytospin 3 centrifuge, Thermo Scientific, Asheville, NC) yielding intact monolayers with an average of around 15,000 single cells.

### Multiplex interphase (mi)FISH hybridization

**Figure 1:**
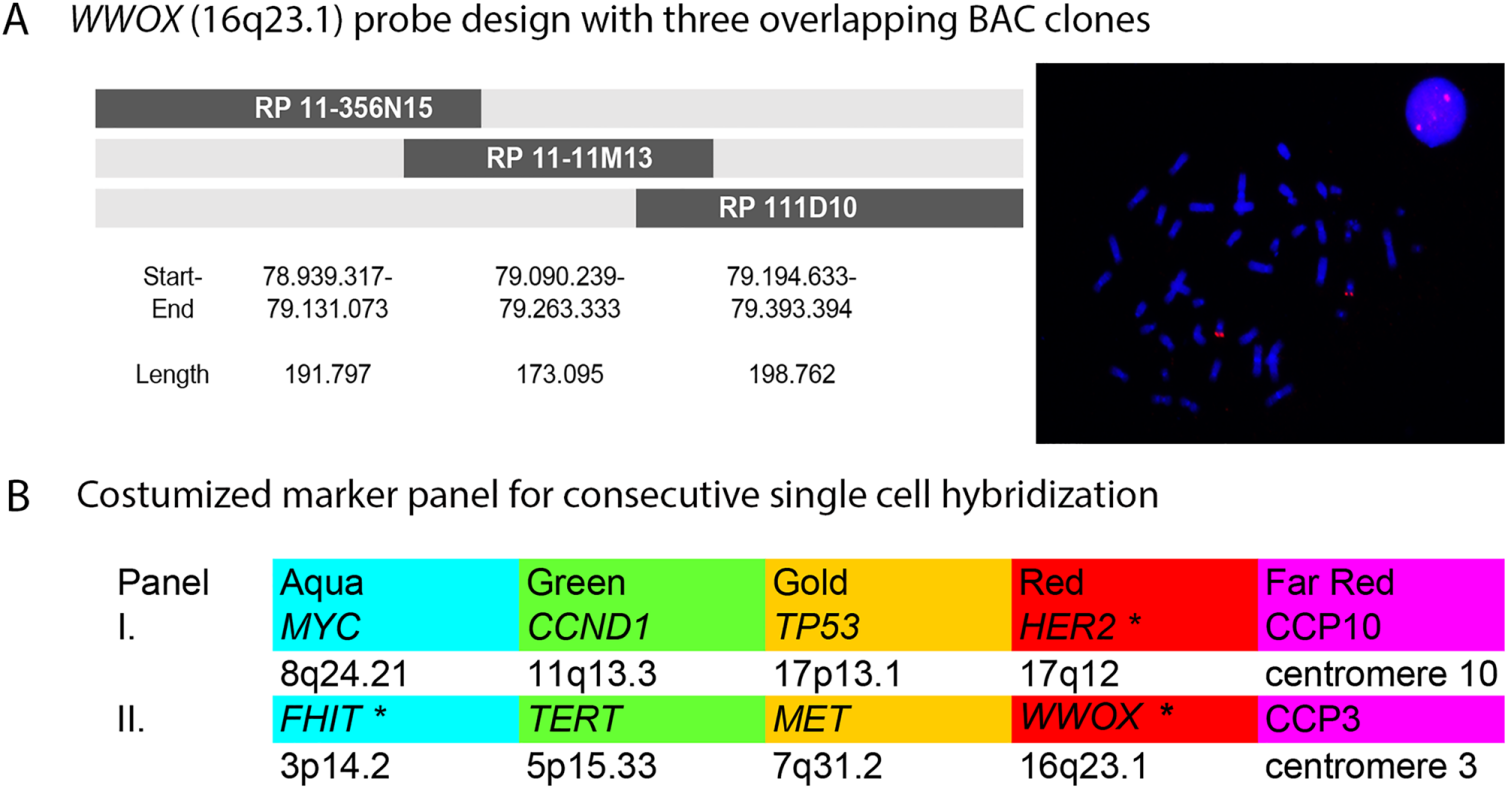
(**A**; left side) Scheme of overlapping *WWOX* BAC clones, which are subsequently combined to create the *WWOX* probe, showing clone name, start, end and length of clone. Clones were fluorescently labeled by nick translation and tested for correct location and signal strength and background before they were combined. (**A**; right side) Lymphocyte cell and metaphase spread hybridized with BAC clone for the *WWOX* probe. Signals appear within interphase nucleus and in metaphase spreads on chromosome arm 16q consistent with the locus for the *WWOX* gene. (**B**) Complete marker panels with indication of color and chromosomal localization. *Stars indicate genetic markers that have previously not been investigated in NASH-HCC.

Bacterial artificial chromosome (BAC) contigs covering the eight target genes were differentially labeled and combined into two panels (Fig. 1). A novel *WWOX* probe, covering the FRAD16 fragile site was designed for this study, extracting and testing BAC clones for the *WWOX* contig before the finalized *WWOX* probe was labeled and combined with the other, already established probes^31,32^. All probes were custom ordered (Cytotest, Rockville, MD). The contigs for *MYC* and *FHIT* were labeled in Aqua (Dyomics 415), for *CCND1* and *TERT* in Green (Dyomics 505), for *TP53* and *MET* in Gold (Dyomics 547P1), and for *HER2* and *WWOX* in Red (Dyomics 590)^33^. Probes for the centromeres of chromosomes 3 and 10 were labeled in Far Red (Dyomics 651) and added to the panels as ploidy control probes. The cytospins were pepsin treated (0.01%, 2 min) denatured in 70% formamide/2 × standard saline citrate for 90 s on a ThermoBrite StatSpin System (Abbott Molecular, Inc.), dehydrated and air-dried. Probe panels were denatured (5 min, 73 °C) and preannealed (1 h, 37 °C). 2 µl probe panel per slide were added to each cytospin, covered with a 12 mm^2^ round coverslip and sealed with rubber cement. The slides were hybridized in a humid chamber (37 °C) for 18–48 h, and detected as previously described in Ref.^31^.

### Imaging and counting

The slides were scanned for 12,000 nuclei using a fluorescence microscope (Olympus BX-63, Tokyo, Japan) equipped with custom optical filters (Chroma, Bellow Falls, VT, USA) with an automated stage and custom scanning and analysis software (DUET/SOLO acquisition and analysis software version 3.7.2.5 https://bioview.com/ BioView Ltd., Rehovot, Israel). After scanning, the coverslip was removed and the slides were washed in 2xSSC, stripped in 70%FA/2xSSC at 80 °C for 30–60 s, dehydrated and air-dried. The slides were then re-hybridized with panel 2 prior to a second scan with exact nuclei relocation by the BioView system. The first two scans were checked for hybridization quality for all markers. Markers that did not hybridize sufficiently, were repeated in a third custom-made panel to assure optimal analysis conditions for all the markers. Images were automatically overlaid for the same target nuclei. Nuclei and signal counts for all FISH probes were presented in a gallery overview, which allows for manual correction of the automated counts. A nucleus was excluded from analysis if it overlapped with another nucleus or if the nucleus was damaged or incomplete or not all probe signals were clearly visible. 300 nuclei were reviewed in detail per sample. Only aberrant nuclei (based on FISH signal patterns) were included in the final analysis assuming that diploid/tetraploid cells represent normal liver or stromal cells. A “signal pattern” was defined as the string of actual copy numbers observed for each cell. To plot the cells according to their gained and lost markers (see color charts) we compared signal numbers to cell ploidy. All markers with signal numbers higher than the ploidy are plotted as gained, all markers with signal numbers lower than the ploidy are lost and when signal numbers are the same as the ploidy, the marker is plotted as unchanged or neutral. Instability index was calculated as the number of signal patterns/100 nuclei. A gain and loss pattern observed in more than 2% of the nuclei within any sample was considered clonal (referred to as a “clonal pattern”). For the fatty liver biopsies, 3000 cells were screened first for cell ploidy and consecutively for numerical aberrations.

Signal counts were recorded in Excel spreadsheets that were exported and used for subsequent FISHtrees analysis that implements a ploidy-based tree building method based on mixed integer linear programming (MILP) https://ftp.ncbi.nih.gov/pub/FISHtrees/^34^.

### Sequencing

To test for associations between chromosomal instability and the mutational status of the most frequently alterated HCC driver genes, targeted sequencing of *TERT*, *CTNNB1 and TP53* (exon 5–8) was conducted. The following primer pairs were used: *CTNNB1* (5′-TTCAATGGGTCATATCACAGATTCTT-3′, 5′-GTTCTCAAAACTGCATTCTGACTTTC-3′), *TERT* (5′-GCACAGACGCCCAGGACC-3′, 5′-GCGGGGAGCGCGCGGCAT-3′; and for short fragmented DNA: 5′-TAATACGACTCATCATAGG*AAGAGGAAGGG*CACGTGGCGGAGGGACTG-3′, 5′-GATTTAGGTGACACTATAG*TTGTGGTTGGT*CGCGGAAAGGAAGGGGA-3′ (including a sequence for sequencing primers (underlined) and a spacer (in italics)). *TP53* (exon 5: 5′-CACTTGTGCCCTGACTTT CA-3′, 5′-AACCAGCCCTGTCGTCTCT-3′; exon 6: 5′-GCCTCTGATTCCTCACTGAT-3′, 5′-TTAACCCCTCCTCCCAGAGA-3′; exon 7: 5′-GGCTCCTGACCTGGAGTCTT-3′, 5′-CTCATCTTGGGCCTGTGTTATCTC-3′; exon 8: 5′-GCCTCTTGCTTCTCTTTTCC-3′, 5′-TAACTGCACCCTTGGTCTCC-3′). PCR steps included 95 °C for 1 min, 57 °C for 1 min, 72 °C for 1 min for 40 cycles for *CTNNB1*. For the *TERT* and *TERTshort* amplification the annealing temperature was 63 °C and 56 °C, and for *TP53* 60 °C for all exons. PCR products were analysed by gel electrophoresis and subsequently purified and sequenced on an ABI 3130xL sequencer using Big Dye terminator chemistry (Applied Biosystems). For *CTNNB1* and *TERTshort* the following sequencing primers were used: Betacat_Ex3seq: 5′-TAAAGTAACATTTCCAATC-3′; TERT_LowSeq2: 5′-CTTCCCACGTGCGCAGCA-3′; T7modf: 5′-TAATACGACTATCATAGG-3′ and SP6r: 5′-GATTTAGGTGACACTATAG-3′. For *TP53* sequencing the amplification primers were used. Sequences were analyzed using the Sequencing Analysis Software and the SeqScape Software https://www.thermofisher.com/ch/en/home/life-science/sequencing/sanger-sequencing.html (both Applied Biosystems), using the corresponding reference sequence (*CTNNB1*: NM_001904.3; *TERT*: NM_198253.2, *TP53*: NM_000546.6) and described according to international conventions on cDNA and protein level. Despite the DNA fragmentation typical for archival FFPE material, targeted sequencing produced reliable results for our samples. However, whole genome sequencing approaches could not be pursued.

## Results

### Chromosomal instability as an indicator for intratumor heterogeneity

**Figure 2:**
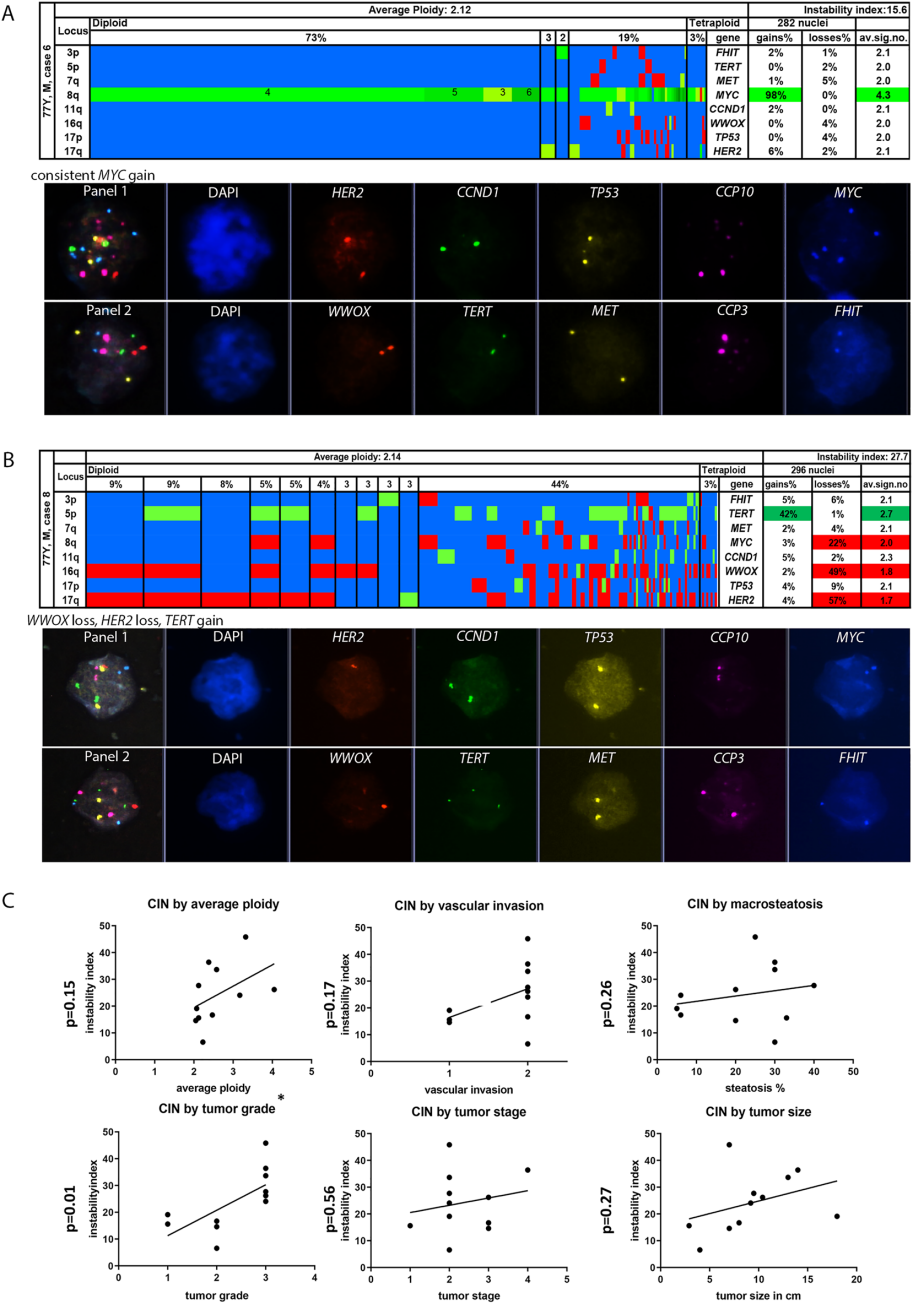
(**A**–**C**) Color display of miFISH analysis with eight gene-specific probes supplemented with a gallery view of signal patterns for representative nuclei in NASH-hepatocellular carcinoma cases. Copy number counts for each nucleus coded as follows: green, gains; red, losses; blue, unchanged. Detail from left to right: the “locus” column shows the chromosome arm the probes reside on. Cells with the same gain and loss patterns are grouped according to frequency and ploidy (vertical lines). The average ploidy (center, centromere signal/number of nuclei) and the instability index (top right, number of signal patterns/100 nuclei.) are calculated from all counted nuclei (296 and 282 respectively). (**A**) HCC case showing consistent *MYC* gain in 98% in diploid and tetraploid tumor cells (color figure) and relatively low instability index of 15.6 (number of signal patterns/100 nuclei). (**B**) HCC case with several tumor subclones, present in diploid and tetraploid cells, high instability index (27.7), due to different signal patterns comprising *HER2*, *WWOX* loss and *TERT* gain. The loss of oxidoreductase *WWOX* and *HER2* gains are potentially associated with NASH-induced hepatocellular carcinoma. DUET/SOLO acquisition and analysis software version 3.7.2.5 https://bioview.com/ (**C**) Correlations of histopathological findings and chromosomal instability index with respective p-values (graphPad Prism software version 9.4.1 https://www.graphpad.com/scientific-software/prism/). *CIN* Chromosomal instability.

**Figure 3:**
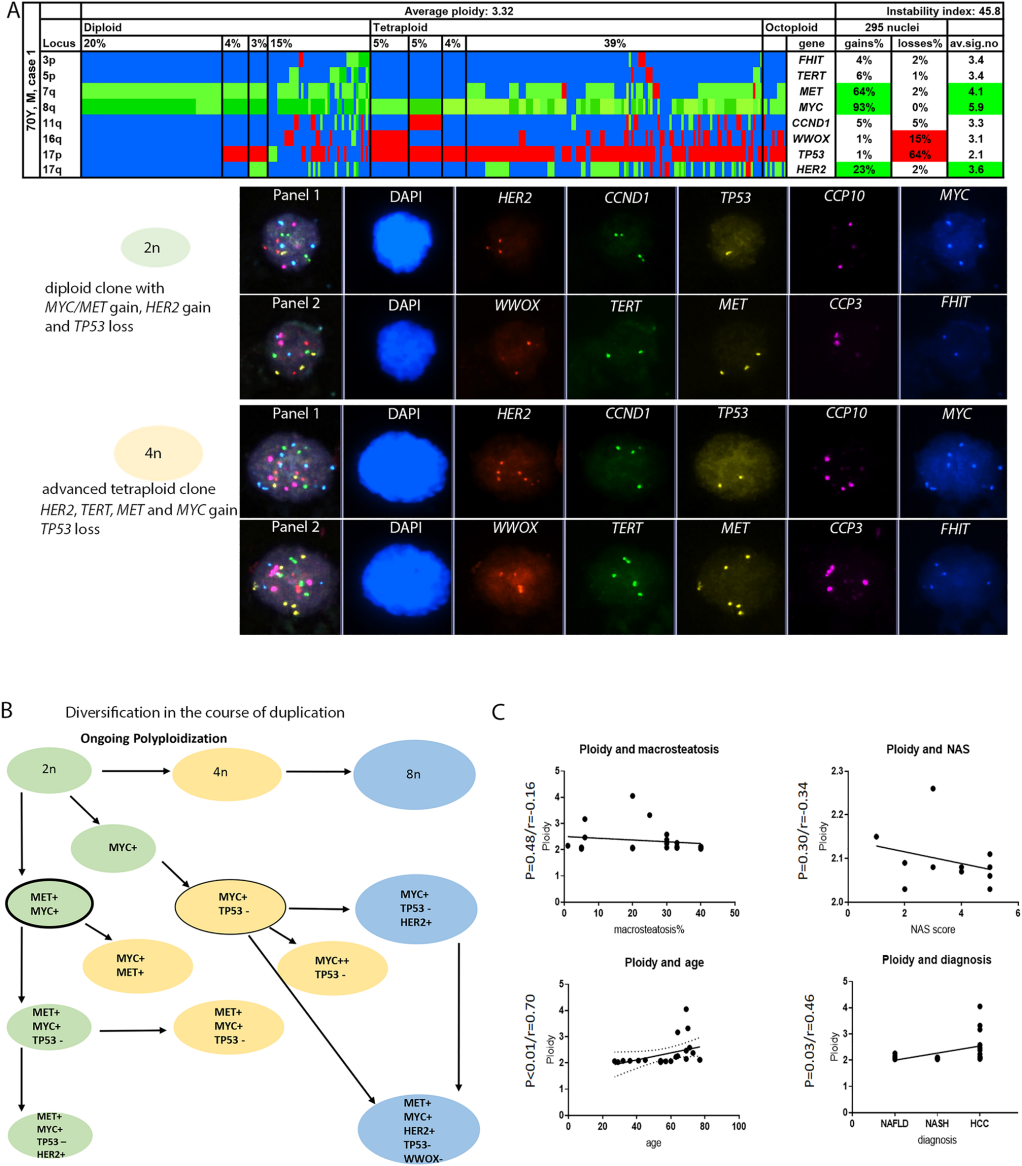
(**A**–**C**) Color display of miFISH analysis with eight gene-specific probes and gallery view summarizing a representative “evolving” nuclei showing genome duplication and additive genomic alterations of case 1. Green, gains; red, losses; blue, unchanged. Detail from left to right: the “locus” column shows the chromosome arm. Average ploidy (center) and percentages of tumor clones (top left to right) are grouped into diploid, tetraploid and octoploid tumor clones. High instability index (45.8), due to many different signal patterns/100 nuclei. (**A**) HCC case 1 with *TP53* loss and *MYC/MET* and *HER2* gain in diploid cells. Tetraploid cells also show *TP53* loss and gains of *MYC*, *MET, HER2 and additionally TERT*. DUET/SOLO acquisition and analysis software version 3.7.2.5 https://bioview.com/. (**B**) Schematic (condensed) tree of case 1, where diversification of tumor cell happens during polyploidization, see full FISH tree in Supplementary Fig. 2. (**C**) Correlations of histopathological findings and ploidy with respective p-and r-values (graphPad Prism software version 9.4.1 https://www.graphpad.com/scientific-software/prism/).

NASH-HCC cases (n = 11) showed intra- and intertumor heterogeneity evidenced by the number of distinct clonal signal patterns (average 8 clonal patterns per tumor, range 5–14; Figs. 2, 3). Observed aberrations were *TP53* loss, *TERT* gain, *MYC* gain, *MET* gain, *WWOX* loss and *HER2* loss/gain with *TP53* loss (5/11; 45%) and *MYC* gain/*WWOX* loss (4/11; 36%) being the most frequent ones. *TP53* mutations were observed to co-occur with *TP53* losses (3/5 cases, 60%) and polyploid tumor clones were found in all three cases. One case with almost exclusively tetraploid clones carried a *TP53* H179R somatic mutation. Three cases carried *CTNNB1* mutations and 9/11 cases revealed *TERT* promoter mutations, but no correlation was found with chromosomal changes.

*TERT* gained cases (3/11; 27%) showed clones with either an additional *MET* gain or additional *HER2* and *WWOX* losses. Of note, two cases showed *HER2* gain (4–6 copies), representing minor or major clones. To assess corresponding protein expression for the *HER2* gains in those two cases, we conducted immunohistochemistry (IHC) evaluation. Neither of the two cases showed positive membranous expression (Supplementary Fig. 1).

HCC cases with a low degree of diversification (4–6 clonal patterns) were comprised of major clones making up to 50–80% of the cell population. Those HCCs characterized by strong major clones were found in n = 4/11 hepatocellular carcinoma cases (Table 1, example Fig. 2A). 7/11 cases showed a high degree of intratumor heterogeneity (Fig. 2B) with up to 14 clonal patterns per case (multiclonality) indicating high chromosomal instability. The calculated instability index ranged from 6.6 to 45.8 with a median of 24 and a mean of 25 ± 11.4. For further analysis, cases were dichotomized along the median. In five cases with a high CIN above 24, tumor grade 3 was diagnosed (p = 0.03). Other histopathological parameters such as tumor size, angioinvasion and higher tumor stage were not significantly correlated with the chromosomal instability, though a positive trend was detected (Fig. 2C). Figure 2C summarizes correlation analysis of histopathological parameters and the instability index. Of the 11 HCC, 9 were classified as NOS (hepatocellular carcinoma, not otherwise specified), and two cases had clear cell subtypes (cases 6 and 9) according to the WHO classification^28^, genotype–phenotype correlation could not be detected (p = 0.57).

### Tumor phylogenetics analysis

By modelling with the tumor phylogenetics software FISHtrees, we found that tumor clones were almost always related to each other, i.e., every clone was either parent or evolved offspring. The modelling of tumor evolution within HCC cases uncovered polyploidization in n = 9/11 cases, 82% (exemplary case, Fig. 3 and Supplementary Fig. 2). We observed two patterns of polyploidization: almost all cases had polyploid nuclei with equivalent numerical aberrations, meaning that the same losses and/or gains were carried over from diploid to tetraploid or even octoploid tumor clones. In three cases, tetraploid or octoploid tumor clones showed not only equivalent numerical aberrations but also had accumulated additional aberrations compared to diploid clones. This means that acquisition of additional chromosomal copy number changes was happening during and/or after polyploidization. The proportion of polyploid compared to diploid tumor clones within each hepatocellular carcinoma varied between 2 and 95% (mean 14.5, Supplementary Fig. 3). FISHtrees did model up to two whole genome doubling events allowing for a ploidy increase from 2 to 8, but did not model a possible third doubling to a ploidy of 16, even though it was (very rarely) observed in the cases.

### Polyploidy in HCC vs. NAFLD/NASH specimens

The average ploidy of HCC cases was 2.7 ± 0.7, ranging from 2.07 to 4.05, the values retrieved from manually counted tumor cells (Table 2). In contrast, in NAFLD tissues the calculated mean ploidy of 2.09 was lower than in HCC (2.09 vs. 2.7; p < 0.05) and slightly higher compared to normal liver tissue (2.05). The count of polyploid hepatocytes within NAFLD lesions yielded an average of 3.9% (range 2.03–10%). The ratio of tetraploid:octoploid cells ranged from 3:1 to 26:1. The correlation of ploidy in NAFLD/NASH specimens with macrosteatosis, the nonalcoholic steatohepatitis score (NAS) showed a trend towards decreasing ploidy, i.e. an inverse association but not a significant correlation (Fig. 3C). Fibrosis was not correlated with polyploidy (p = 0.32). NAFLD/NASH lesions did not show any clonal copy number changes for the miFISH probe panel applied.

**Table 2:** Single-cell ploidy in nonalcoholic fatty liver disease (NAFLD) and nonalcoholic steatohepatitis (NASH) patient specimens.

Case	Age	Diagnosis incl. steatosis^a^	NAS^b^	Proportion tetra:octoploidy	Polyploidy fraction	Ploidy mean
1	41	NAFLD 10%	3	9:1	3.90%	2.09
2	32	NAFLD 20%	3	20:1	2.90%	2.08
3	69	NAFLD, 5% steatosis, incomplete cirrhosis	1	16:1	6.70%	2.15
4	36	NASH, 40% steatosis,	4	18:1	3.80%	2.08
5	27	NASH, 70% steatosis	4	9:1	2.90%	2.07
6	64	NAFLD, 20%	3	5:1	10.00%	2.26
7	54	NAFLD, 20% steatosis, incomplete cirrhosis	2	12:1	1.90%	2.03
8	29	NASH, 70% steatosis	5	26:1	1.80%	2.03
9	45	NASH, 60% steatosis	5	7:1	4.40%	2.11
10	57	NASH, 80% steatosis	5	3:1	2.00%	2.06
11	54	NASH, 30% steatosis	5	9:1	3.50%	2.08
				Mean	3.98%	2.09

^a^Diagnosis with macrosteatosis.

^b^Nonalcoholic steatohepatitis activity score, including three criteria: macrosteatosis, hepatocellular ballooning and lobular inflammation; high scores means more severe disease, i.e. manifest steatohepatitis is defined by a minimum score of 4+.

## Discussion

We undertook the first attempt of a single-cell based copy number analysis looking at polyploidy and aneuploidy in nonalcoholic steatohepatitis (NASH)-induced hepatocellular carcinoma and its precursor lesion nonalcoholic fatty liver disease.

Multiclonality as an indicator for intratumor heterogeneity in hepatocellular carcinoma is not a novel finding and is a cause for many challenges in the treatment of patients with hepatocellular carcinoma^35^. Not only does it bear the problem of sampling errors for diagnosis and prediction, it might also be responsible for modest chemotherapy response^36^. Intratumor heterogeneity goes along with high chromosomal instability and vice versa. In our study, we were able to show that this concept holds true on a single-cell level and for the specific subgroup of NASH-induced HCC. The question of whether chronic liver diseases and liver cirrhosis lead to multiregional tumor development (comparable to a field effect) or whether the HCC progress evolves from single tumor clones is unresolved. Interestingly the single-cell results in our study show that most tumor clones are related to each other and their numerical aberrations progress from parental to further evolved clones, which was also shown in a study using genomic and methylation profiles indicating an evolutionary process starting from a single event^37^. A study by Zhai et al. observed two different tree topologies, consisting of either related or deeply separated clades^38^. The authors propose that phylogenetic inference decreases with distance to the tumor center. Our phylogenetic findings for the HCC samples mirror the trunk-branch-model proposed by Swanton and his colleagues originally described for kidney cancer^39^. HCC can be therefore considered as a tumor with branched evolution and several levels of complexity, implying that therapy resistance may increase with each level of complexity. One limitation of our study is, of course, the selected 8-marker panel and the low number of cases.

Polyploidization was recently proposed as a biomarker for poor prognosis in hepatocellular carcinoma^19^. Our single-cell data support why this is likely: we found that polyploidization via endoreplication/genome duplication is ongoing while numerical aberrations in hepatocytes emerge and accumulate as shown by our FISHtrees analysis. This implies tetraploidization as a risk factor for developing chromosomal instability^25^. Supportive of this concept, recent studies on di-ethylnitrosamine treated mice have demonstrated that centrilobular hyperploidization give rise to preneoplastic lesion formation^40^. The relationship between *TP53* loss and tetraploidization might be crucial in this setting. Interestingly, in mouse models it was shown that tetraploid, but not diploid cells give rise to tumors when p53 is inactivated^41^. Conversely, polyploidy allows the cells to compensate for the effects of loss of heterozygosity of tumor suppressor genes. Altogether, balancing polyploidy might be a matter of the timepoint: in chronic liver disease, tetraploidy acts tumor-suppressive^27^ and as a buffer against genomic damage with a potential for genetic variation and adaptation as described in Duncan’s theory of the ploidy conveyor^24,25^. In ongoing liver tumorigenesis, ploidy bears an increased risk for genomic instability. Via mutational events, cells are prone to loss of heterozygosity (LOH)^20^. As possible mechanism how proliferative polyploid cells give rise to tumors (among *TP53* mutations) ploidy reduction was suggested, which impairs the gatekeeper function of polyploidy and aggravate LOH^26^. Although in our data we clearly see accumulated, i.e. quantitavely more alterations in polyploid cells, which speaks against an “upward” tumor evolution from polyploid cells back to diploid cells. With regards to whole-chromosome (polysomy) or arm-level-changes as reason for observed amplification, FISHtrees modeling can distinguish whole chromosome gains/losses from more localized aberrations for chromosomes 3 and 17 since they were represented with two probes each within the panel, but for other chromosomes with a single probe it cannot. However, we have observed that also higher copy numbers, which are more likely to be based on focal amplifications, are often faithfully doubled during polyploidization (unless extrachromosomal DNA, like double minutes, is the basis for the amplification).

We observed losses of *WWOX,* the fragile site marker, in 4 cases (36%). Interestingly, monosomy of chromosome 16, where *WWOX* resides, was reported in mice as being protective against toxins^24^. In chronic liver disease models, *WWOX* loss was among the initial alterations preceding morphological visible neoplastic changes^42^. In HCC cell line studies, loss of *WWOX* copy numbers was observed and correlated well with absent or lower mRNA expression^18^. Our data show that *WWOX* loss could also be important in HCC from patients with NASH etiology. Genome wide analysis of copy number variations (CNV) combined with gene expression profiling, as has been conducted on hepatitis B virus-related HCC^43^, could help to further evaluate the relevance of *WWOX* loss and other copy number changes in NASH-induced HCC. These studies would require testing a new cohort with available fresh frozen tumor samples, because the FFPE samples do not preserve quality of the DNA or RNA well enough to perform bulk sequencing.

*HER2* gains were previously reported to be of low frequency in HCC (2.42% by immunohistochemistry and 0.1% by FISH)^44^, compared to 18% on single-cell level in our study. Whether this finding is due to a technical advantage of the single-cell FISH or whether *HER2* alterations might be truly driver enriched in NAFLD associated HCC requires further analysis.

Increased polyploidy preceeding hepatocarcinogenesis in nonalcoholic fatty liver specimens was previously reported. In a study by Gentric et al. the fraction of highly polyploid mononuclear cells reached 16% and 18% in NASH patients with and without concomitant HCC compared to a control group of chronic liver disease and HCC of other etiologies^45^. Interestingly, Gentric et al. also stated that polyploidy was independent from severity of fibrosis. In our study, we also could not find a correlation of ploidy and fibrosis or other NAFLD/NASH criteria (steatosis, NAS score). Our mean fraction of polyploidy (in biopsy specimens) was 4% (max. 10%) and not significantly increased in NAFLD/NASH patients compared to healthy liver donors of different ages because the single-cell preparation does only take into account mononuclear polyploidy (as a result of endoreplication) and not bi-nucleated hepatocytes (dominant mechanism cytokinesis failure). NAFLD/NASH lesions of mild/borderline stages (n = 5) and of severe/intermediate stages (n = 6) did not show any clonal copy number changes for the miFISH probe panel applied, indicating that these lesions were not likely to be driven by copy number changes in the selected genes. Mechanisms for the progression of NAFLD/NASH to liver cancer remain incompletely understood, pathogenesis could involve DNA damage of other loci, inflammatory response, genetic modifiers as *PNPLA3* or *TM6SF2* or mutations such as the recently reported *ACVR2A* mutations^6,46^.

The miFISH technology, similar to single-cell sequencing, works with intact single cells, which in this study were derived from disintegrated archival tissue samples from a 4 cm^2^ area per tumor. The accurate evaluation of exact copy number clones, especially when multiplexing ten probes is only possible using intact, non-overlapping nuclei that can be visually inspected for completeness and hybridization efficiency. Truncation artifacts, overlapping nuclei and suboptimal hybridizations that are intrinsic to tissue FISH are detrimental to clonal reconstruction and phylogenetic FISHtrees modeling. However, the tissue disintegration, which is needed for good quality preparation as the backbone of the miFISH technology, has the drawbacks of losing tissue architecture and spatial resolution. That means that peritumoral spatial resolution was not obtained and in NAFLD/NASH biopsy specimens, liver zonation responsible for liver specific functions as detoxification or synthesis, could not be studied. However, zonation (higher amount of centrilobular polyploidy) was only found in mice, and not in human tissues^20,40^.

In summary, miFISH single-cell analysis in NASH-induced hepatocellular carcinoma further refined the relationship of genomic instability and polyploidy, showing tumor evolution via genome duplication during hepatic oncogenesis. The loss of the fragile site marker *WWOX* and *HER2* gains are novel findings potentially associated with NASH-induced hepatocellular carcinoma.

## Supplementary Information

Supplementary Information.

## Data Availability

The data are available at https://www.ncbi.nlm.nih.gov/genbank accession numbers OP480193-OP480212.
