# Supplementary material for: Single-cell resolved ploidy and chromosomal aberrations in nonalcoholic steatohepatitis-(NASH) induced hepatocellular carcinoma and its precursor lesions
Source: Sci Rep. 2022 Dec 31;12:22622. doi: 10.1038/s41598-022-27173-z (PMC9805444; doi:10.1038/s41598-022-27173-z)
Supplement: Supplementary file 1 — Supplementary Information. [file 41598_2022_27173_MOESM1_ESM.pdf]

Supplemental material to:

Single-Cell resolved Ploidy and Chromosomal Aberrations in Nonalcoholic Steatohepatitis-(NASH) Induced Hepatocellular Carcinoma and its Precursor Lesions.

Juliane Friemel, Irianna Torres, Elizabeth Brauneis, Tim Thörner, Alejandro A. Schäffer, E. Michael Gertz, Tobias Grob , Kati Seidl , Achim Weber, Thomas Ried, Kerstin Heselmeyer-Haddad

Legends:

Supplementary Figure 1 (A-C) Color display of miFISH analysis with eight gene-specific probes and gallery view showing a case with *HER2* amplifikation. Chart: Green, gains; red, losses; blue, unchanged. **(A)** Detail from left to right: the „locus" column shows the chromosome arm. Average ploidy (center) and percentages of tumor clones (top left to right) are grouped into diploid, tetraploid and octoploid tumor clones. **(B)** *HER2* signals varied between 4-6 copies/cell, and 8-11 copies in tretraploid cells. Additional *MYC* gains and *TP53* loss. **(C)** Immunohistochemistry staining for HER2 showed no specific membranous staining.

Supplementary Figure 2 Original FISH tree of case 1. The string of numbers represent the copy numbers of genes as follows: *FHIT-TERT-MET-MYC-CCND1-WWOX-TP53-HER2*. Size of ovals represents resprective clone frequency. Root is a normal diploid clone.

Supplementary Figure 3 Color display of miFISH analysis of all cases that are not shown in Figure 2 and 3. Green, gains; red, losses; blue, unchanged. Detail from left to right: the „locus" column shows the chromosome arm. Average ploidy (center) and percentages of tumor clones. Instability index: signal patterns/100 nuclei.

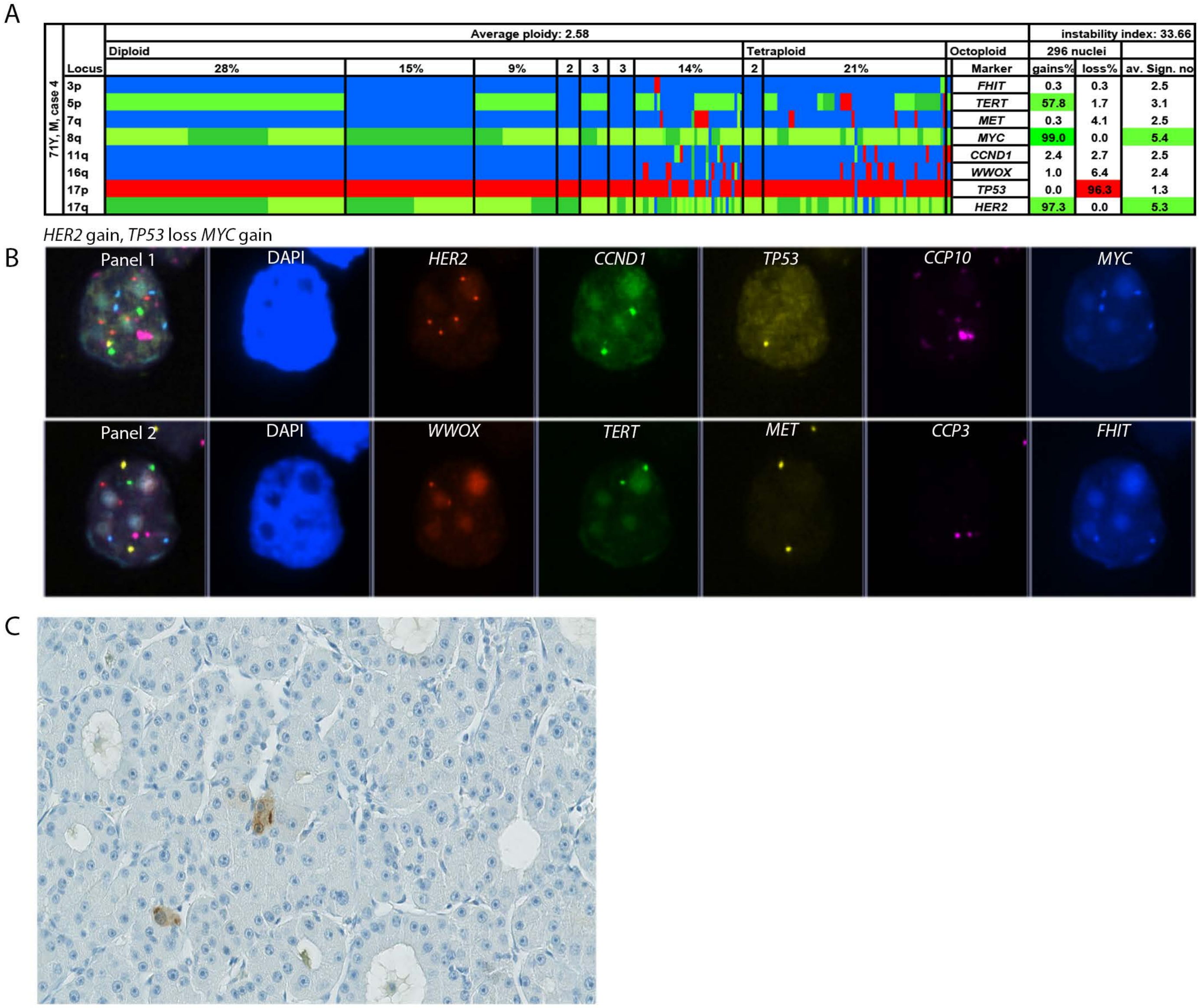
A
71Y, M, case 4
Average ploidy: 2.58
instability index: 33.66
Diploid
Tetraploid
Octoploid
296 nuclei
Locus
28%
15%
9%
2
3
3
14%
2
21%
Marker
gains%
loss%
av. Sign. no
3p
5p
7q
8q
11q
16q
17p
17q
FHIT 0.3 0.3 2.5
TERT 57.8 1.7 3.1
MET 0.3 4.1 2.5
MYC 99.0 0.0 5.4
CCND1 2.4 2.7 2.5
WWOX 1.0 6.4 2.4
TP53 0.0 96.3 1.3
HER2 97.3 0.0 5.3
HER2 gain, TP53 loss MYC gain
B
Panel 1
DAPI
HER2
CCND1
TP53
CCP10
MYC
Panel 2
DAPI
WWOX
TERT
MET
CCP3
FHIT
C

Supplementary Figure 2

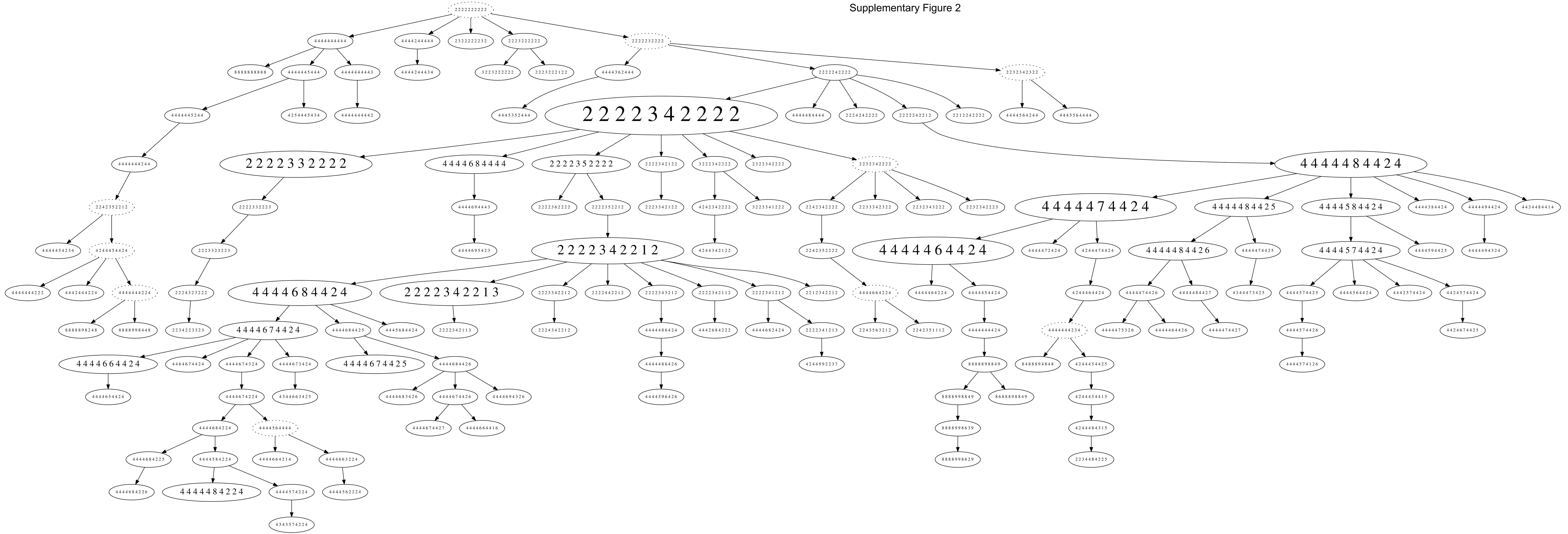

Supplementary Figure 3

69Y, M, case 2 — Average ploidy: 4.05 — Instability index: 26.17 — 298 nuclei

Dip 4% | Tetraploid 32% | 33% | 5% | 5% | 4% | | 32% | Octoploid

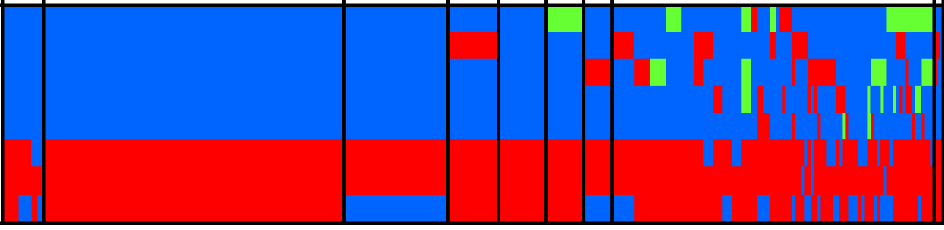

Locus	gene	gains%	losses%	av.Sig.no
3p	*FHIT*	10	2	4.1
5p	*TERT*	0	14	3.8
7q	*MET*	6	10	3.9
8q	*MYC*	3	5	3.9
11q	*CCND1*	1	3	3.9
16q	*WWOX*	0	93	2.1
17p	*TP53*	0	99	2.0
17q	*HER2*	0	74	3.1

74, M case 3 — Average ploidy: 2.38 — Instability index: 36.4 — 250 nuclei

Diploid 14% | 12% | 8% | 7% | 5% | 4 | 3 | 32% | Tetraploid 14%

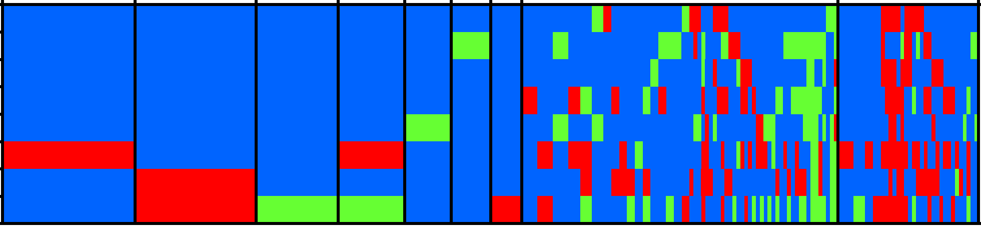

Locus	gene	gains%	losses%	av.Sig.no.
3p	*FHIT*	3	8	2.5
5p	*TERT*	16	4	2.9
7q	*MET*	3	6	2.5
8q	*MYC*	7	11	2.7
11q	*CCND1*	13	3	2.7
16q	*WWOX*	44	3	2.3
17p	*TP53*	2	26	2.3
17q	*HER2*	26	24	2.8

74, M case 3 — Average ploidy: 2.38 — Instability index: 36.4 — 250 nuclei

Diploid 14% | 12% | 8% | 7% | 5% | 4 | 3 | 32% | Tetraploid 14%

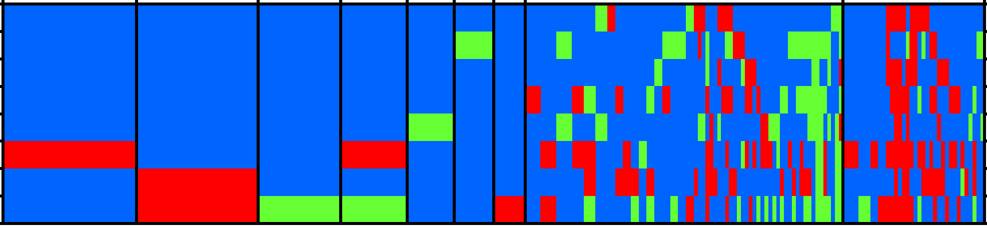

Locus	Gene	gains%	losses%	av.Sig.no.
3p	*FHIT*	3.2	7.6	2.5
5p	*TERT*	16	3.6	2.9
7q	*MET*	2.8	6	2.5
8q	*MYC*	7.2	11	2.7
11q	*CCND1*	13.2	3.2	2.7
16q	*WWOX*	44	3.2	2.3
17p	*TP53*	2	26	2.3
17q	*HER2*	26	24	2.8

60Y, M, case 7 — Average ploidy: 2.07 — instability index: 19.11 — 293 nuclei

Diploid 33% | 17% | 5% | 4% | 3 | 3 | 3 | 2 | 2 | 2 | 23% | Tetraploid

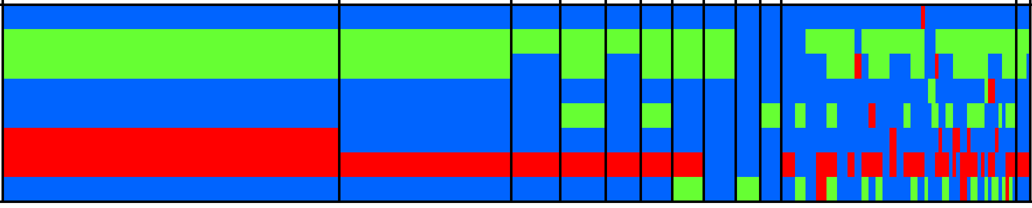

Locus	gene	gains%	losses%	av. Sig.no
3p	*FHIT*	0	0	2.1
5p	*TERT*	91	0	3.8
7q	*MET*	78	1	3.0
8q	*MYC*	1	1	2.0
11q	*CCND1*	17	1	2.2
16q	*WWOX*	0	35	1.8
17p	*TP53*	0	84	1.5
17q	*HER2*	13	2	2.2

64Y, F, case 9 — Average ploidy: 3.17 — Instability index: 24.08 — 191 nuclei

Diploid 25% | 10% | 6% | 4% | 4 | 4 | 3 | 3 | 3 | 2 | 2 | 11% | Tetraploid 7% | 4 | 13% | 2

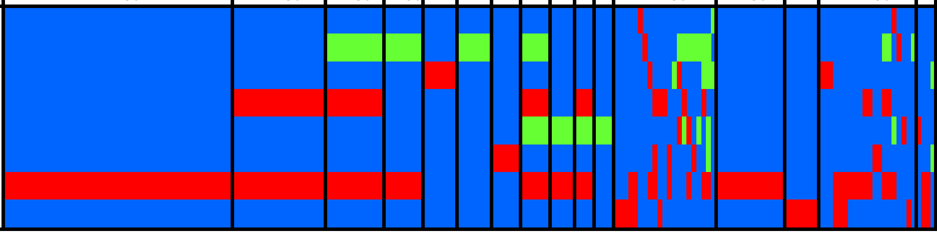

Locus	gene	gains%	losses%	av. Sign.
3p	*FHIT*	0	1	2.9
5p	*TERT*	23	1	3.2
7q	*MET*	3	6	3.1
8q	*MYC*	0	26	2.8
11q	*CCND1*	12	2	3.1
16q	*WWOX*	1	6	3.0
17p	*TP53*	0	71	2.4
17q	*HER2*	0	1	2.8

62Y, M, case 10 — Average ploidy: 2.23 — Instability index: 6.55 — 290 nuclei

Diploid 79% | 3 | 3 | 7% | Tetra 7% | Octoploid

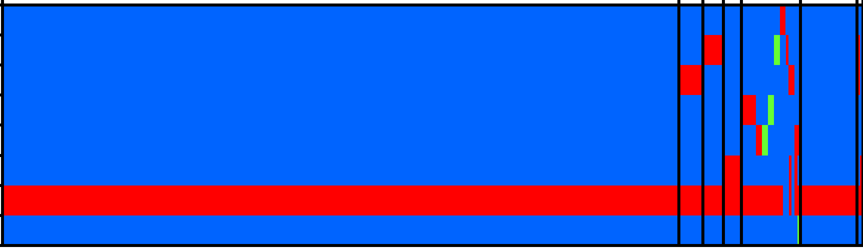

Locus	gene	gains%	losses%	av. Sig. no
3p	*FHIT*	0	1	2.1
5p	*TERT*	1	3	2.1
7q	*MET*	0	4	2.1
8q	*MYC*	1	2	2.1
11q	*CCND1*	1	1	2.1
16q	*WWOX*	0	3	2.1
17p	*TP53*	0	99	1.1
17q	*HER2*	0	0	2.1

54Y, M, case 11 — Average Ploidy: 2.08 — Instability Index: 14.2 — 300 nuclei

70.0% | 13.3% | | 14.0%

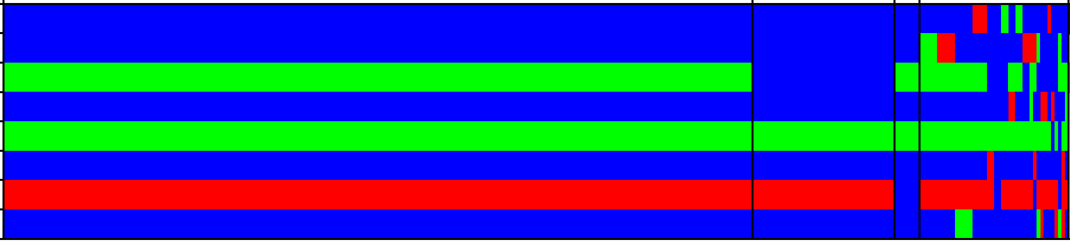

Locus	gene	gains%	losses%	AvgSig
3p	*FHIT*	1%	2%	2.1
5p	*TERT*	2%	3%	2.1
7q	*MET*	82%	0%	2.9
8q	*MYC*	1%	2%	2.1
11q	*CCND1*	99%	0%	9.4
16q	*WWOX*	0%	1%	2.1
17p	*TP53*	0%	96%	1.1
17q	*HER2*	2%	1%	2.1
